# Effect of *Lactiplantibacillus* and sea buckthorn pomace on the fermentation quality and microbial community of paper mulberry silage

**DOI:** 10.3389/fpls.2024.1412759

**Published:** 2024-08-30

**Authors:** Shun Peng, Lingling Xie, Yuyao Cheng, Qiqi Wang, Li Feng, Yang Li, Yonghui Lei, Yanfei Sun

**Affiliations:** ^1^ College of Life Sciences/Xinjiang Production and Construction Corps Key Laboratory of Oasis Town and Mountain-basin System Ecology, Shihezi University, Shihezi, Xinjiang, China; ^2^ Department of Plant Protection, College of Agriculture, Shihezi University, Shihezi, Xinjiang, China

**Keywords:** *Lactiplantibacillus*, *Weissella*, silage, paper mulberry, sea buckthorn pomace

## Abstract

**Background:**

Paper mulberry is a promising alternative fodder source due to its high protein and the abundance of active components. However, paper mulberry often faces susceptibility to contamination during silage fermentation, and there is a need to improve the quality of silage fermentation of paper mulberry through exotic additives. Sea buckthorn pomace (BP) is a feed additive containing antimicrobial and antioxidant substances that help to enhance silage fermentation. Therefore, the objective of this study was to evaluate the effects of BP and *Lactiplantibacillus* as additives on silage fermentation and bacterial community of paper mulberry.

**Results:**

The results showed that BP and *Lactiplantibacillus* significantly reduced the pH and ammonium nitrogen content of paper mulberry silage (*P* < 0.05) and significantly increased the content of lactic acid and acetic acid (*P* < 0.05), resulting in more residual water-soluble carbohydrate and crude protein contents and less fiber content relative to the control. The key microorganisms in paper mulberry silage fermentation are *Lactiplantibacillus pentosus* and *Weissella cibaria*. Among these, *Lactiplantibacillus* favored a rapid increase in *Lactiplantibacillus pentosus* abundance during the pre-silage fermentation period, whereas BP favored the promotion of *Lactiplantibacillus pentosus* growth, resulting in higher contents of lactic and acetic acid than those of the control.

**Conclusions:**

Simultaneously adding *Lactiplantibacillus* and BP can effectively improve the quality of paper mulberry silage and increase the abundance of beneficial microorganisms in paper mulberry silage.

## 1 Introduction

Due to the rapid development of animal husbandry in China, traditional feeds, such as fodder crops, pasture, and grains, have proven insufficient to meet the burgeoning demands of animal husbandry. One potential solution to the problem of feed shortage is the development of new feed sources. In recent years, extensive research has been dedicated to woody forages in China. Paper mulberry (*Broussonetia papyrifera* L.), a dioecious tree native to East and Southeast Asia, has garnered attention for its richness in crude protein (CP) (Guo et al., 2019). Paper mulberry is rich in crude fat, phenolic compounds, flavonoids, terpenes, and glycosides, while maintaining low fiber levels (Hao et al., 2021; Zhang et al., 2022). Furthermore, paper mulberry displays considerable resilience to low temperatures, drought, and infertile soil conditions. Consequently, paper mulberry represents a promising new source of feed (Yu et al., 2023). Previous studies have confirmed the efficacy of supplementing the diets of dairy cows, beef cattle, fattening pigs, and goats with paper mulberry to reduce feeding costs, enhancing livestock production performance while fortifying livestock immunity (Hao et al., 2021; Hua et al., 2020). However, the presence of low water-soluble carbohydrate (WSC) content, low lactic acid bacteria (LAB) population, and high leaf buffering capacity in paper mulberries leads to slow silage initiation, long fermentation time, and easy contamination of proteins with stray bacteria (Zhang et al., 2022). Additionally, paper mulberry contains anti-nutritional factors such as tannin, which can form precipitates by binding with enzymes, sugars, proteins, and metal ions in animal feed, resulting in a decline in the digestion and absorption rates of nutrients and a reduction in the feed’s nutritional value (Cheng et al., 2021; Guo et al., 2021). In response to this situation, various improvement measures have been implemented, and diverse additives have been investigated to enhance the preservation of nutrients in paper mulberry. For example, Cheng et al. (2022) found that the addition of LAB was able to reduce the number of molds and Enterobacter in paper mulberry silage and reduce the breakdown of CP. Wu et al. (2022) also found that the addition of a low proportion of maize meal was effective in improving the quality of paper mulberry silage fermentation.

Sea buckthorn (*Hippophae rhamnoides* L.) is a cultivated plant with significant economics and medicament widely distributed in Xinjiang China (Ciesarová et al., 2020; Rousi, 1971). The fruits and seeds of sea buckthorn are rich in nutrients, including unsaturated fatty acids, vitamins, carotenoids, phenolic compounds, flavonoids, triterpene enol, isoprenoid alcohols, and other essential elements with antimicrobial and antioxidant properties that contribute to silage preservation (Gâtlan and Gutt, 2021). Numerous studies have shown that the simultaneous addition of BP and LAB to silage can effectively increase the abundance of beneficial microorganisms such as *Lactiplantibacillus*, *Lactococcus*, and *Leuconostoc* and reduce contamination with harmful microorganisms (Chen et al., 2020). This is mainly due to the organic acids produced by LAB, which acidify the cytoplasm of harmful microorganisms by releasing H^+^, leading to physiological structural damage and the death of harmful microorganisms. The flavonoids contained in BP can inhibit the synthesis of harmful microbial nucleic acids, suppress energy metabolism, alter cell membrane permeability, and lead to the death of harmful microorganisms. In addition, BP can also provide a large amount of WSC to LAB, leading to the production of large amounts of LA by LAB, which can lower the silage pH, create an acidic environment, and prevent the decomposition of crude protein and the production of ammonium nitrogen (NH_3_-N). These functions are of great significance for improving the fermentation performance of mulberry silage feed.

The objective of this study was to investigate the effects of *Lactiplantibacillus* and BP on the fermentation quality, nutrient composition, and microbial structure of paper mulberry silage. Furthermore, the potential of *Lactiplantibacillus* and BP to address the challenges associated with pH decline, protein deterioration, and microbial contamination in paper mulberry silage was explored.

## 2 Materials and methods

### 2.1 Silage preparation

On 22 June 2023, paper mulberry (Hybrid paper mulberry 201) was harvested from the experimental field in Kuitun City, Xinjiang Uygur Autonomous Region (a temperate desert arid climate, 44.43°N, 84.92°E, altitude: 392.6 m). The test material was the first harvest of the paper mulberry (4-5 harvests can be cut throughout the year). The paper mulberry was harvested at a height of approximately 140 cm, and only the leaves of the paper mulberry were harvested at the time of harvest. The BP utilized in this study was obtained from a plantation located in Shawan City, Xinjiang Uygur Autonomous Region, China. The chemical composition of paper mulberry and BP are shown in **Table 1**. The harvested paper mulberry was cut into 1-2 cm lengths and divided into four blocks to obtain 18 replications per treatment (six storage periods × three replicates). The treatments were as follows: (1) control (CK), 2 ml distilled water; (2) BP, at an optimal application rate of 5% fresh material (FM); (3) LP, *Lactiplantibacillus*, isolated from natural fermented paper mulberry silage, at a recommended application rate of 10^5^ cfu/g FM (4) LP + BP, BP (applied at 5% of FM) and *Lactiplantibacillus* (applied at 1×10^5^ cfu/g of FM). Then, according to the method of Li et al. (2022), approximately 300 g (average 168.13 kg DM/m^3^) of chopped paper mulberry was mixed homogenously with each additive, packed manually into polyethylene bags (25 cm × 30 cm), and then vacuum packed using a vacuum packing machine (V-79, Shenzhen Dimo Technology Industry Co., Ltd.). Three bags for each treatment were opened and sampled after 1, 3, 7, 14, 30, 60 d of ensiling, respectively. A total of 72 samples (4 treatments × 18 replicates) were prepared for analysis of fermentation quality, chemical composition, and bacterial community composition.

**Table 1:** Chemical composition of fresh paper mulberry and sea buckthorn pomace.

Items	Paper mulberry	Sea buckthorn pomace	SEM	*P*-value
DM (% FM)	28.48b	93.78a	0.01	< 0.001
CP (% DM)	29.59a	19.93b	1.30	< 0.001
WSC (% DM)	13.07b	16.26a	0.49	< 0.001
NDF (% DM)	38.57a	32.73b	0.09	< 0.05
ADF (% DM)	22.12a	20.77b	0.08	0.288
pH	6.73a	3.38b	0.10	< 0.001

Values with different letters in the same row are significantly different (P < 0.05). FM, fresh material; DM, dry matter; CP, crude protein; WSC, water-soluble carbohydrate; NDF, neutral detergent fiber; ADF, acid detergent fiber; SEM, standard error of the mean.

### 2.2 Chemical composition analysis

The silage sample (20 g) was suspended in sterile water (180 ml) within a 250 ml conical flask. Subsequently, the flask was tightly sealed and stored in a refrigerated environment at 4°C for a duration of 24 h, and then filtered through eight layers of cheesecloth. The filtrate was subjected to centrifugation (12,000 × g, 10 min, 4°C). The pH of the resulting filtrate was then assessed using a Remco pH-3E meter (Bernardes et al., 2019). The determination of NH_3_-N employed a phenol hypochlorite colorimetric method, while the quantification of soluble sugars utilized an anthrone-sulphuric acid colorimetric method (Broderick and Kang, 2019). Additionally, the concentrations of LA, acetic acid (AA), propionic acid (PA), and butyric acid (BA) in the filtrate were analyzed using high-performance liquid chromatography (Zhang et al., 2016).

The dry matter (DM) of the silage was assessed by drying approximately 5 g of silage samples in an oven at 105°C for 4 h until a constant weight was attained. The silage was subsequently dried, crushed, and ground to pass through a 40-mesh screen. It was then analyzed for CP, neutral detergent fiber (NDF), acid detergent fiber (ADF), and WSC. The total nitrogen (TN) content was determined using a Kjeldahl apparatus (HR-1000, Shanghai Lichen Instrument Technology Co., Ltd), and CP was calculated by multiplying TN by 6.25 (AOAC, 1990). ADF and NDF content was assessed using the methods described by Van Soest et al. (1991) by using a Fiber Analyzer (ANKOM 2000, ANKOM Technology Corp). WSC was determined by the anthrone colorimetric method of Murphy (1958).

### 2.3 Bacterial community analysis

The genomic DNA of the silage was extracted using a TGuide S96 Magnetic Stool DNA Kit (Tiangen Biotech (Beijing) Co., Ltd.) according to the manufacturer’s instructions. The full-length 16S ribosomal RNA (rRNA) gene was amplified using a specific primer (27F and 1492R) with the barcode. The amplicons were quantified, after which the normalized equimolar concentrations of amplicons were pooled and sequenced on the PacBio Sequel II platform (Beijing Biomarker Technologies Co., Ltd., Beijing, China).

The bioinformatics analysis of this study was performed with the aid of the BMKCloud (http://www.biocloud.net/). The raw reads generated from sequencing were filtered and demultiplexed using the SMRT Link software (version 8.0) with the minPasses ≥5 and minPredictedAccuracy ≥ 0.9, in order to obtain the circular consensus sequencing (CCS) reads. Subsequently, the lima (version 1.7.0) was employed to assign the CCS sequences to the corresponding samples based on their barcodes. CCS reads containing no primers and reads beyond the length range (1,200–1,650 bp) were discarded through the recognition of forward and reverse primers and quality filtering using the Cutadapt (version 2.7) quality control process (Bolger et al., 2014). The UCHIME algorithm (v8.1) was used to detect and remove chimera sequences to obtain clean reads (Martin, 2011). Sequences with similarity > 97% were clustered into the same operational taxonomic unit (OTU) by USEARCH (v10.0), and OTU counts less than 2 in all samples were filtered (Edgar, 2013).

The qualifying sequences with more than 97% similarity thresholds were allocated to one OTU using USEARCH (version 10.0). Taxonomy annotation of the OTUs was performed based on the Naive Bayes classifier in QIIME2 using the SILVA database (release 138.1) with a confidence threshold of 70% (Bolyen et al., 2019; Christian et al., 2013). Alpha was performed to identify the complexity of species diversity of each sample utilizing QIIME2 software. Beta diversity calculations were analyzed using principal coordinate analysis (PCoA) to assess the diversity in the samples for species complexity. One-way analysis of variance (ANOVA) was used to compare bacterial abundance and diversity. The online platform BMKCloud (https://www.biocloud.net) was used to analyze the sequencing data.

### 2.4 Statistical analysis

All statistical analyses were performed using SPSS version 22.0 (IBM Corp., Armonk, NY, United States). The characteristic data of paper mulberry silage were analyzed using ANOVA. Significant differences between treatments were determined using Tukey’s test at *P* < 0.05.

## 3 Results

### 3.1 Chemical composition of the paper mulberry silage

In the process of ensiling paper mulberry, there were significant changes in chemical composition, and these changes ultimately affected the quality of paper mulberry silage. As shown in **Table 2**, the addition of BP and *Lactiplantibacillus* significantly influenced the DM of paper mulberry silage. At 60 d, the DM content of the LP + BP (28.25% FM) group was significantly higher than others (*P* < 0.05). At 60 d, the WSC of the LP + BP (8.65% DM) group was the highest, followed by the BP (7.24% DM, *P* < 0.05) group. The CP content of each group was significantly higher than that of the control (*P* < 0.05).

**Table 2:** Effects of *Lactiplantibacillus* (LP), sea buckthorn pomace (BP), and LP + BP on the chemical composition of silage after 60 days of ensiling.

Items	CK	BP	LP	LP + BP	SEM	*P*-value
DM (% FM)	23.73d	26.93b	25.38c	28.25a	0.46	< 0.001
DM LOSS (% FM)	5.38a	2.19c	3.74b	0.87d	0.46	< 0.001
WSC (% DM)	6.16b	7.24b	6.32b	8.65a	0.53	< 0.05
CP (% DM)	20.73b	22.33ab	22.87a	22.82a	0.86	< 0.05
NDF (% DM)	42.52	43.64	39.88	40.13	2.52	0.311
ADF (% DM)	25.49a	23.30b	23.92ab	22.97b	0.74	< 0.05

Values with different letters in the same row are significantly different (P < 0.05). CK, control (paper mulberry only); BP, paper mulberry with sea buckthorn pomace; LP, paper mulberry with *Lactiplantibacillus*; LP + BP, paper mulberry with sea buckthorn pomace and *Lactiplantibacillus*. DM, dry matter; FM, fresh material; DM LOSS, dry matter loss; WSC, water-soluble carbohydrate; CP, crude protein; NDF, neutral detergent fiber; ADF, acid detergent fiber; SEM, standard error of the mean.

**Figure 1:**
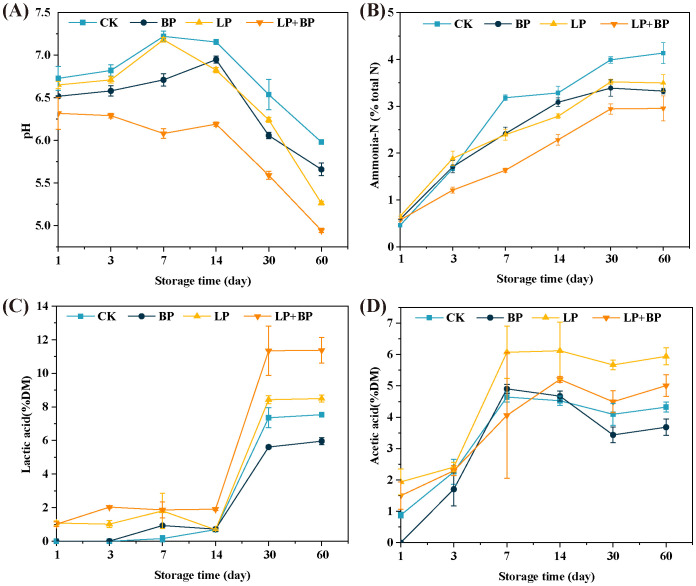
The effects of *Lactiplantibacillus* (LP), sea buckthorn pomace (BP), and LP + BP on pH **(A)**, ammonium nitrogen **(B)**, lactic acid **(C)**, and acetic acid **(D)** of paper mulberry silage.

The changes in pH, NH_3_-N, LA, and AA during paper mulberry silage fermentation are shown in **Figure 1** (the supplementary data are presented in **Supplementary Table S1**.). As shown in **Figures 1A, B**, the pH value decreases, and the NH_3_-N content increases during the silage fermentation process. At 60 d, the pH value and NH_3_-N content of each group were significantly lower than the control, with LP + BP having the lowest pH value (4.94) and NH_3_-N content (2.96% DM), indicating that the addition of BP and *Lactiplantibacillus* were both effective in reducing the decomposition of CP during paper mulberry silage. The presence of organic acids is an important criterion for determining the acceptability of silage. As shown in **Figures 1C, D**, PA and BA were not detected in all groups of silage. At 60 d, the LA content of the LP + BP group was significantly higher than that of the others and the AA content of the BP was significantly higher than that of the others (*P* < 0.05).

### 3.2 Bacterial community diversity in the paper mulberry silage

**Figure 2:**
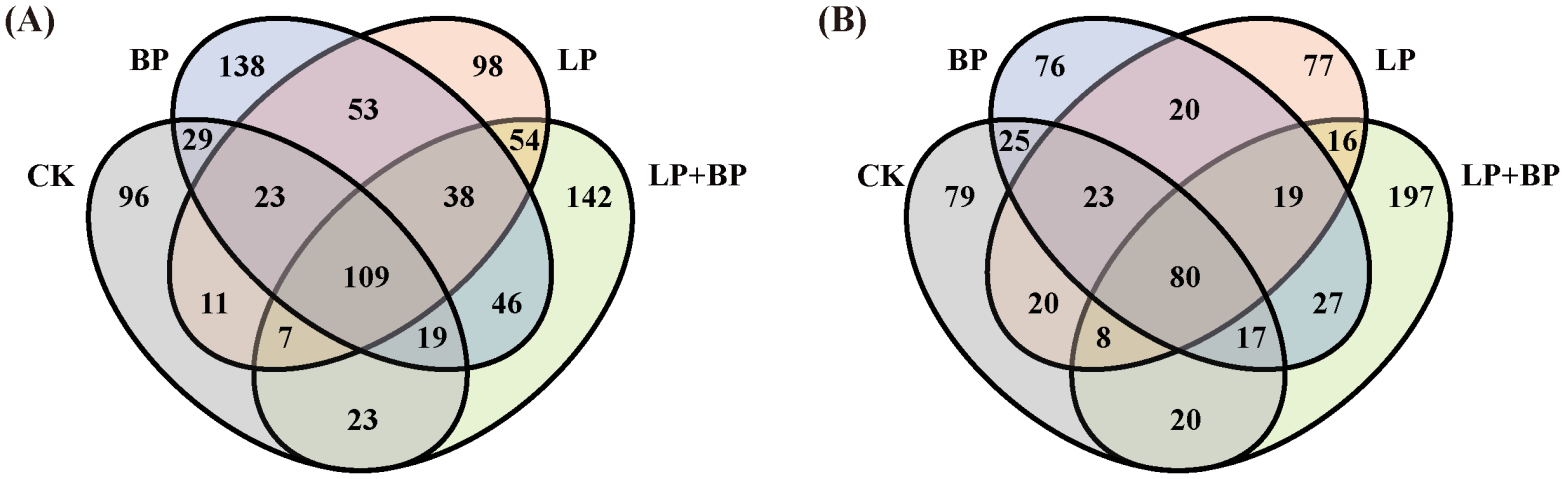
Venn analysis of OTUs in silage fermentation of paper mulberry at 97% sequence identity [**(A)** Venn diagram representing 1d of fermentation; **(B)** Venn diagram representing 60 d of fermentation].

**Figure 3:**
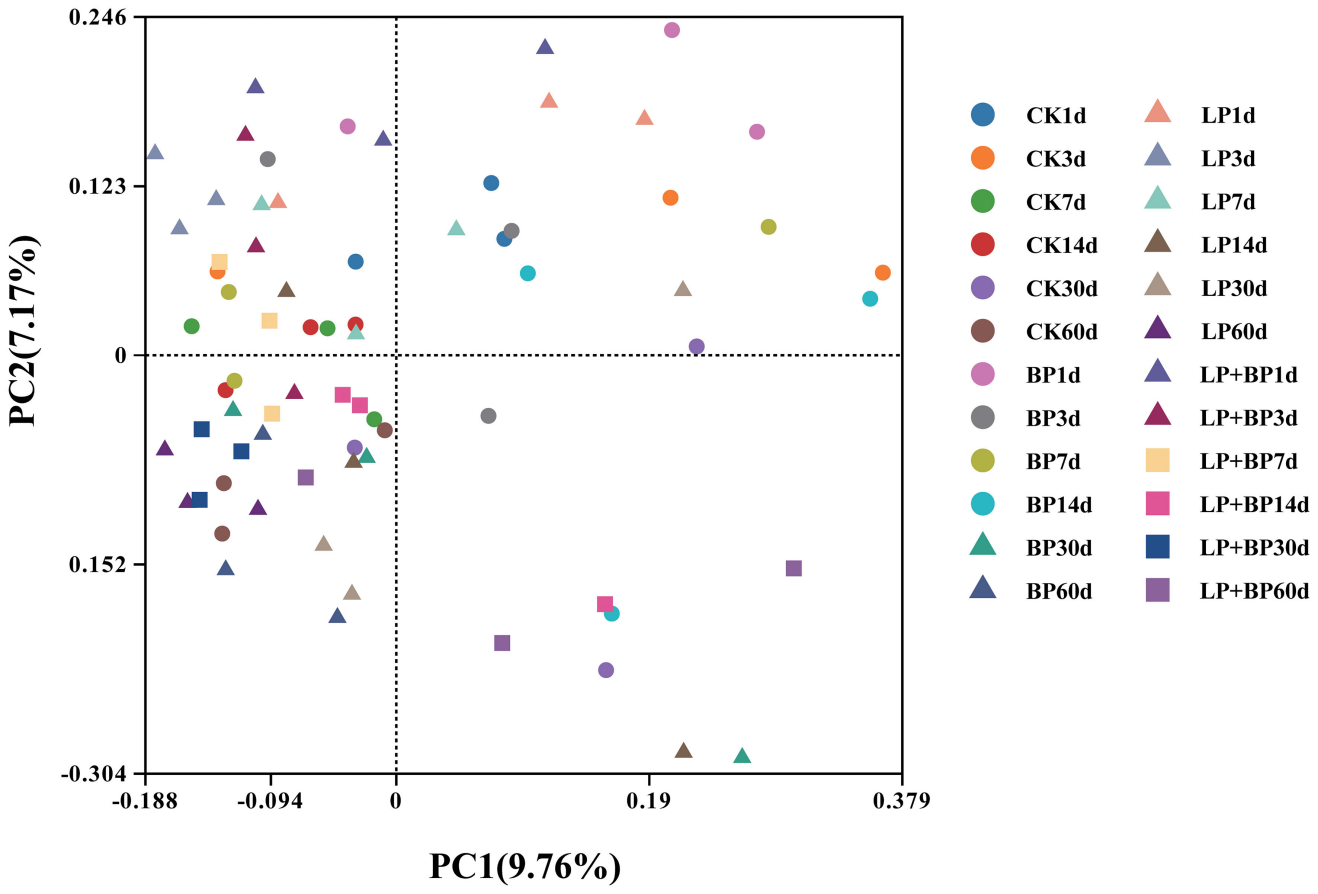
The community dissimilarities in different treatments and fermentation time, calculated by unweighted UniFrac distances, with coordinates calculated by principal coordinates analysis.

On average, each sample yielded 57,443 CCS sequences through full-length sequencing of bacterial 16S rRNA amplicons in silage. During the silage fermentation process, the microbial community undergoes significant changes. As shown in **Figure 2**, silage fermentation led to a decrease in the OTUs across all bacterial groups. However, it is worth noting that the OTUs of unique bacteria in LP+BP increased. **Figure 3** shows significant disparities in bacterial species among silages with different fermentation times. During the ensiling process, distinct clusters were observed at two distinct time intervals: 1-3 d and 7-30 d. After 60 d of ensiling, there was significant variability in microbial species between the LP+BP group and the other silage groups. This suggests that LP + BP had a different microbial community to the other treatments, which may explain the better fermentation of LP + BP.

**Figure 4:**
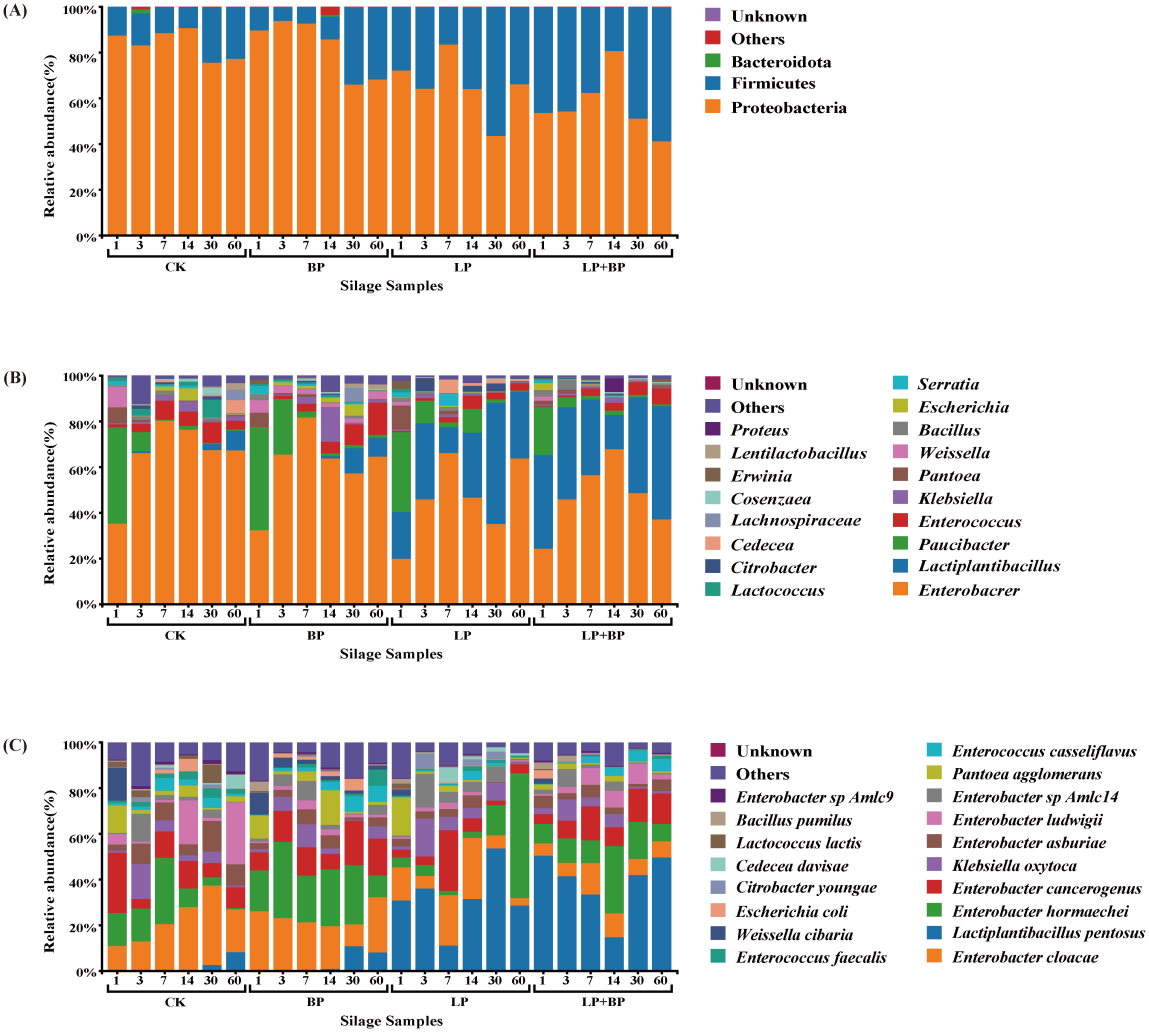
Differences and diversity of microbial communities in paper mulberry silage [**(A)** relative abundance of paper mulberry silage bacterial phylum across different treatments and fermentation times, **(B)** relative abundances of paper mulberry silage bacterial genus across different treatments and fermentation time, **(C)** relative abundances of paper mulberry silage bacterial species across different treatments and fermentation time].

In the ensiling process, the alterations in bacterial communities are depicted in **Figure 4**. The primary representatives of dominant groups in the fermentation of various silage feeds are Proteobacteria and Firmicutes (**Figure 4A**). **Figure 4B** illustrates the changes in the bacterial community at the genus level during the fermentation process. The prevalent species in the silage included *Lactiplantibacillus* and *Enterobacter*. Throughout the fermentation process, *Enterobacter* dominated in CK and BP, while the abundance of *Lactiplantibacillus* gradually increased on 14 d. In the LP and LP + BP groups, as fermentation progressed, the abundance of *Enterobacter* gradually decreased, while the abundance of *Lactiplantibacillus* increased. However, in the LP and LP + BP groups, the abundance of *Lactiplantibacillus* both exhibited a brief decrease on 7 and 14 d. By 60 d, the content of *Lactiplantibacillus* in the LP + BP group was significantly higher than in the others. The results at the species level were similar to those at the genus level (**Figure 4C**). *Lactiplantibacillus pentosus* in LP and LP + BP had relatively high abundance at the beginning of silage. Although there was a decline in *Lactiplantibacillus pentosus* in the LP and LP + BP groups during fermentation, at 60 d, the abundance of *Lactiplantibacillus pentosus* was significantly higher in the LP and LP + BP groups than in CK and BP.

### 3.3 Random forest analysis of characteristic bacteria in paper mulberry silage

**Figure 5:**
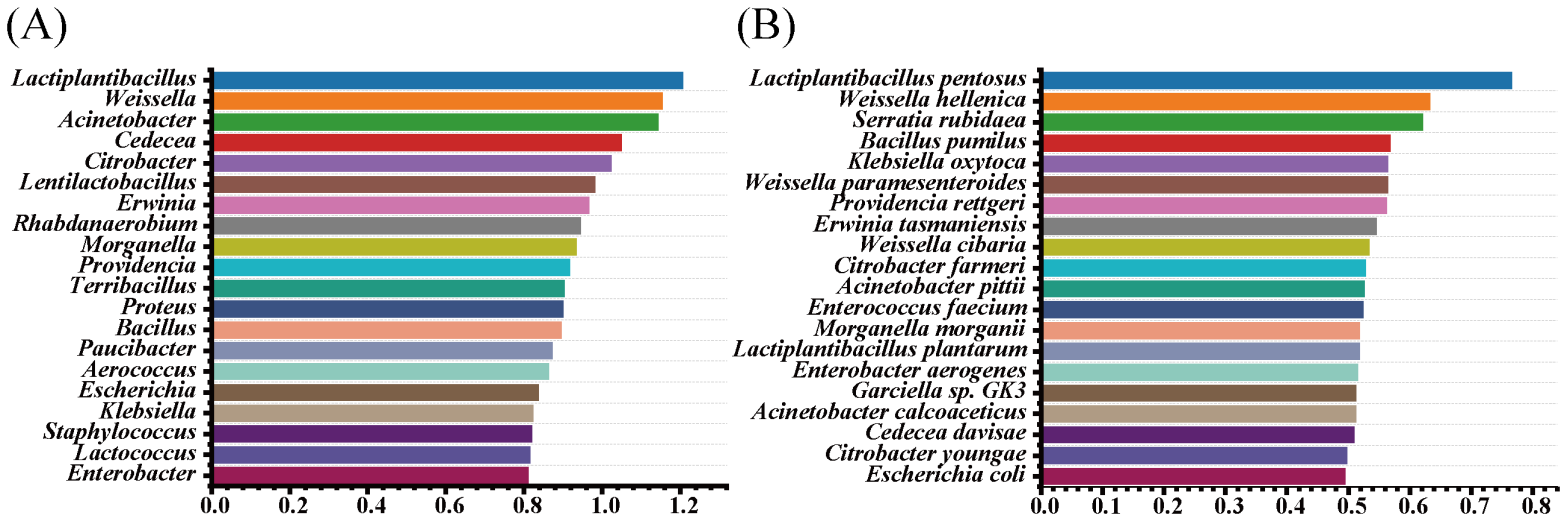
Random forest model of effect sizes on the bacterial community of the most abundant taxa of difference before and after silage of paper mulberry [**(A)** random forest modeling at the genus level; **(B)** random forest models at the species level].

Random forest classification was used to identify bacterial taxa during silage fermentation, aiming to discover biomarkers. From **Figure 5A**, it can be seen that the main microorganisms responsible for the observed differences among the groups at the genus level were *Lactiplantibacillus*, *Weissella*, *Acinetobacter*, *Cedecea*, *Citrobacter*, and others. Noteworthy among them were *Lactiplantibacillus*, *Weissella*, and *Acinetohacter*, identified as the major characteristic genera. At the species level (**Figure 5B**), *Lactiplantibacillus pentosus* and *Weissella helleniea* stood out as the major characteristic species.

### 3.4 Relationship between main species and silage quality

**Figure 6:**
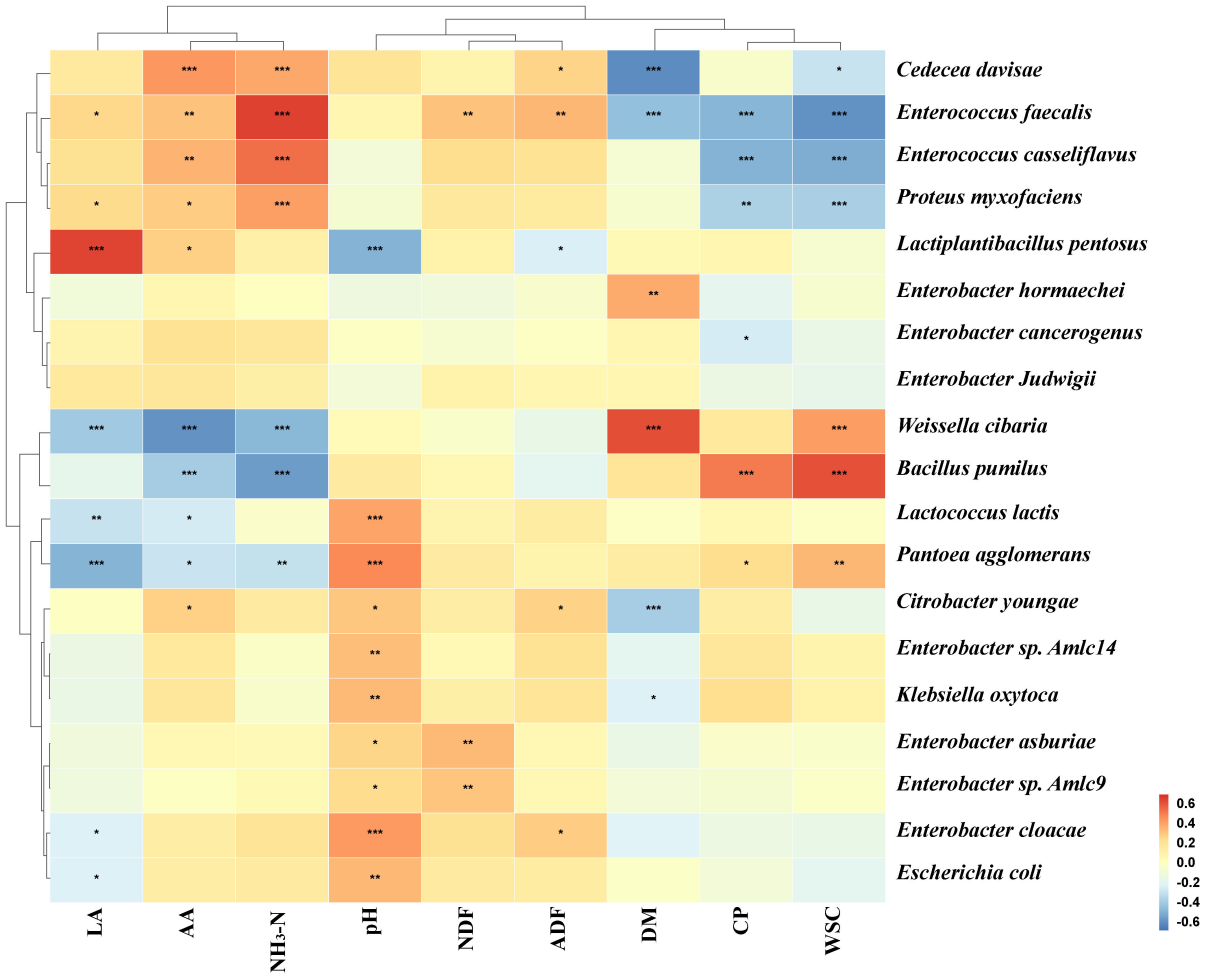
Spearman’s correlation analysis of silage fermentation parameters and the relative abundance of the top 20 species (* is < 0.05, ** is < 0.001, *** is < 0.0001).

**Figure 6** analyzes the Spearman’s correlation between fermentation parameters and kinetics for the initial 20 species in the paper mulberry silage process. The results showed that LA was positively correlated to *Lactiplantibacillus pentosus* and pH was negatively correlated to *Lactiplantibacillus pentosus*. This indicates that *Lactiplantibacillus pentosus* is capable of reducing the pH of paper mulberry silage through the production of LA. CP and WSC were positively correlated to *Bacillus pumilus* and negatively correlated to *Enterococcus faecalis*, *Enterococcus casseliflavus*, and *Proteus myxofaciens*. The correlation between NH_3_-N and bacteria was in opposition to the correlation between CP and bacteria, indicating that NH_3_-N production is predominantly due to the decomposition of CP.

## 4 Discussion

### 4.1 Chemical properties of paper mulberry silage

LAB dominate fermentation during the silage process and reduce pH by producing a large amount of LA (Wang S. et al., 2019). Coblentz and Muck (2012) demonstrated that LA is mainly produced by LAB through fermentation of WSC. In this study, the WSC of 13.07%DM of fresh paper mulberry was sufficient for LAB fermentation. However, the pH (7.22 to 5.98) and LA (at 60 d, 7.53) of the control silage sustained high levels during the process of ensiling. This may be attributed to the fact that the CK lacked LAB. Hao et al. (2021) found that paper mulberry has a strong buffering capacity, leading to difficulties in decreasing the pH of paper mulberry silage. For paper mulberry, a silage fermentation pH of 4.3-5.00 can be considered indicative of excellent silage (Kung et al., 2018). While the LP + BP group exhibited a fermentation pH of 4.94, falling within the good quality range (4.30 < 4.94 < 5.00) at 60 d. This suggests that both BP and *Lactiplantibacillus* were effective in reducing silage pH and improving silage fermentation quality. During the process of silage fermentation, microorganisms convert WSC not only to LA but also to a range of organic acids, including AA, PA, BC, and others. In this investigation, PA and BA were absent during the fermentation process, indicating favorable fermentation conditions for the silage in each group. This favorable result may be attributed to the presence of several bioactive compounds in paper mulberry and sea buckthorn pomace, including flavonoids, lignin, polysaccharides, and terpenoids, which exhibit antibacterial and antioxidant properties (Han et al., 2016; Ciesarová et al., 2020). Consequently, the inhibitory effect on *Acidipropionibacterium* and *Clostridium* prevents the fermentation of sugars into PA and BA, as well as the conversion of LA to PA and BA (Kung et al., 2018).

The production of NH_3_-N is primarily attributed to the decomposition of CP by harmful microorganisms (Kung et al., 2004). A reduction in pH can effectively suppress the growth of harmful microorganisms in silage (Ni et al., 2017; Lu et al., 2022). This is the reason why the NH_3_-N content of the BP, LP, and LP + BP groups was significantly lower than that of CK. The addition of BP increased the polyphenol content of silage. Polyphenols are able to form complexes with proteins, thus hindering protein breakdown (Chen et al., 2020).

### 4.2 The role of micro-organisms in silage

In this study, the OTU levels in the BP and LP+BP groups were higher in the pre-silage period due to the fact that BP has a higher WSC content, which promotes the growth of aerobic bacteria. This is in agreement with the findings of Du et al. (2022). After 60 d, the OTU levels across all groups witnessed a decline. This decline is attributed to the anaerobic and acidic environment established by *Lactiplantibacillus*, suppressing the growth of microorganisms and diminishing their abundance. This suggests that the simultaneous introduction of *Lactiplantibacillus* and sea buckthorn pomace is advantageous for creating a distinctive and favorable fermentation environment in paper mulberry ensilage. Silage is accompanied by complex microbiological changes, different bacteria play different roles in the fermentation process, and usually the most important species in the silage fermentation process are LAB. LAB produce large amounts of LA, which rapidly lowers the silage pH and reduces the harmful microbial composition of the silage, thus improving the nutritional quality and fermentation characteristics in the silage (Ávila et al., 2014). However, in both the LP and LP + BP groups, there was a transient decrease in the abundance of *Lactiplantibacillus* at 7 and 14 d. This decrease can be attributed to a significant presence of *Enterobacter* during the silage fermentation period, converting the LA produced by *Lactiplantibacillus* into AA. As AA is less acidic than LA, it results in an elevated pH of the silage feed, disrupting the growth environment for *Lactiplantibacillus* (Ávila and Carvalho, 2020; Bai et al., 2022). *Lactiplantibacillus* abundance was lower in the CK and BP groups and this was not solely attributable to the absence of added *Lactiplantibacillus*. It is also likely to have been influenced by the high abundance of *Enterobacter* in the CK and BP groups. Prior research has indicated that certain strains of *Enterobacter* can produce limited quantities of acids, such as LA, AA, and succinic acid. Nevertheless, a significant proportion of *Enterobacter* metabolizes LA to other compounds, leading to elevated pH levels in silage and disrupting the environment in which *Lactiplantibacillus* can grow (Santos et al., 2016). During fermentation, *Enterobacter* and *Lactiplantibacillus* also compete for fermentation substrates and convert CP to NH_3_-N (Wang Y. et al., 2019). This may also explain the lower CP content and higher NH_3_-N content in the CK group at the fermentation endpoint. Whereas, the lower NH_3_-N content of BP may be due to the presence of large amounts of *Weissella*, which is usually present in fresh forages and produces a large amount of acid in the early stages of silage fermentation to create a proper acidic environment for the fermentation of LAB (Bai et al., 2022; Sturino, 2018).

Metabolites produced during silage fermentation affect bacterial communities, and metabolites can also be improved by microbial diversity, which affects silage quality (Li et al., 2022). The present study found that *Lactiplantibacillus pentosus* was positively correlated with LA and negatively correlated with pH. This suggests that *Lactiplantibacillus pentosus* is the key microorganism for good silage fermentation. The correlation between NH_3_-N and bacteria was the opposite of the correlation between CP and bacteria. This indicates that NH_3_-N is mainly caused by bacterial decomposition of CP. It is noteworthy that *Weissella cibaria*, despite exhibiting a negative correlation with LA, exhibited a positive correlation with DM and WSC. This indicates that although *Weissella cibaria* is associated with a reduction in silage LA, it may be a pivotal microorganism for the preservation of silage nutrients. Maślak et al. (2022) also discovered that *Weissella cibaria* exhibits robust antimicrobial properties and favorable fermentation characteristics, making it a prospective microorganism for enhanced fermentation. Consequently, it can be postulated that the combination of *Weissella cibaria*, *Lactiplantibacillus*, and BP as a paper mulberry silage additive may result in enhanced fermentation outcomes.

## 5 Conclusion

The bacterial community composition underwent significant changes during the fermentation of paper mulberry silage. Both *Lactiplantibacillus* and BP had a positive impact on the silage quality. The microbial species responsible for fermentation differed among the treatments, reducing species diversity, and increases in LA and a reduction of pH resulted in high-quality fermentation. Specifically, BP as a silage additive showed potential for improving the silage quality of paper mulberry by enriching with *Lactiplantibacillus* during ensiling. *Lactiplantibacillus* inoculation simplifies the microbial community structure, rapidly establishes an acidic fermentation environment, and reduces nutrient losses. Overall, based on the chemical composition, fermentation profile, and bacterial community of silage, our results have confirmed the feasibility of inoculating with *Lactiplantibacillus* and adding BP to produce high-quality silage from paper mulberry.

## Data Availability

Sequence data of this project have been deposited in the Sequence Read Archive (SRA) of the National Center for Biotechnology Information (NCBI) under the accession number: PRJNA1071816 (https://www.ncbi.nlm.nih.gov/bioproject/PRJNA1071816/).

## Supplementary material

Supplementary Table 1 Changes in pH, ammonium nitrogen, lactic acid, and acetic acid during the silage process of paper mulberry. Means with different letters in the same row **(A–F)** or column (a–d) are significantly different (*P* < 0.05). CK, control (paper mulberry only); BP, paper mulberry with sea buckthorn pomace; LP, paper mulberry with *Lactiplantibacillus*; LP + BP, paper mulberry with sea buckthorn pomace and *Lactiplantibacillus*; NH_3_-N, ammonium nitrogen; LA, lactic acid; AA, acetic acid.
