# Supplementary material for: Effect of Lactiplantibacillus and sea buckthorn pomace on the fermentation quality and microbial community of paper mulberry silage
Source: Front Plant Sci. 2024 Aug 30;15:1412759. doi: 10.3389/fpls.2024.1412759 (PMC11395498; doi:10.3389/fpls.2024.1412759)
Supplement: Supplementary Table 1 — Changes in pH, ammonium nitrogen, lactic acid, and acetic acid during the silage process of paper mulberry. Means with different letters in the same row (A–F) or column (a–d) are significantly different (P < 0.05). CK, control (paper mulberry only); BP, paper mulberry with sea buckthorn pomace; LP, paper mulberry with Lactiplantibacillus; LP + BP, paper mulberry with sea buckthorn pomace and Lactiplantibacillus; NH3-N, ammonium nitrogen; LA, lactic acid; AA, acetic acid. [file Table1.docx]

Table S1 Changes in pH, ammonium nitrogen, lactic acid, and acetic acid during the silage process of paper mulberry

| Items | Treatment | Ensiling time (d) | | | | | | SEM |
| --- | --- | --- | --- | --- | --- | --- | --- | --- |
|  |  | 1 | 3 | 7 | 14 | 30 | 60 |  |
| pH | CK | 6.73aBC | 6.82aB | 7.22aA | 7.15aB | 6.64aC | 5.98aD | 0.07 |
|  | BP | 6.52abC | 6.58cC | 6.71bB | 6.95bA | 6.06cD | 5.66bE | 0.05 |
|  | LP | 6.65aD | 6.71bC | 7.18aA | 6.82cB | 6.24bE | 5.26cF | 0.03 |
|  | LP + BP | 6.32bA | 6.29dA | 6.08cB | 5.87dC | 5.59dD | 4.94dE | 0.06 |
| NH_3_-N  （% TN） | CK | 0.46cD | 1.67bC | 3.18aB | 3.19aB | 3.99aA | 4.16aA | 0.09 |
|  | BP | 0.59bE | 1.71abD | 2.42bC | 3.08aB | 3.39abA | 3.32bA | 0.10 |
|  | LP | 0.66aE | 1.89aD | 2.39bC | 2.79bB | 3.52aA | 3.50bcA | 0.09 |
|  | LP + BP | 0.57bE | 1.21cD | 1.64cC | 2.28cB | 2.95bA | 2.96cA | 0.10 |
| LA (% DM) | CK | 0.00bC | 0.00cC | 0.16bC | 0.69bB | 7.35bA | 7.54cA | 0.09 |
|  | BP | 0.00bE | 0.01cE | 0.93abC | 0.72bD | 5.61cB | 5.96dA | 0.07 |
|  | LP | 1.08aBC | 1.02bBC | 1.81aB | 0.68bC | 8.43bA | 8.49bA | 0.32 |
|  | LP + BP | 1.02aB | 2.03aB | 1.87aB | 1.91aB | 11.34aA | 11.37aA | 0.49 |
| AA (% DM) | CK | 0.88bD | 2.26aC | 4.64aA | 4.53bAB | 4.10bB | 4.32dAB | 0.22 |
|  | BP | 0.00cD | 1.71aC | 4.91aA | 4.67bA | 3.44cB | 3.68cB | 0.22 |
|  | LP | 1.94aB | 2.41aB | 6.07aA | 6.12aA | 5.67aA | 5.94aA | 0.46 |
|  | LP + BP | 1.50aB | 2.29aB | 4.06aA | 5.21bA | 4.49bA | 5.01bA | 0.57 |

Means with different letters in the same row (A–F) or column (a–d) are significantly different (*P* < 0.05). CK: control (paper mulberry only); BP: paper mulberry with sea buckthorn pomace, LP: paper mulberry with *Lactiplantibacillus*; LP + BP: paper mulberry with sea buckthorn pomace and *Lactiplantibacillus*. NH_3_-N: ammonium nitrogen; LA: lactic acid; AA: acetic acid.
